# Spatial adiabatic passage of ultracold atoms in optical tweezers

**DOI:** 10.1126/sciadv.adl1220

**Published:** 2024-10-02

**Authors:** Yanay Florshaim, Elad Zohar, David Zeev Koplovich, Ilan Meltzer, Rafi Weill, Jonathan Nemirovsky, Amir Stern, Yoav Sagi

**Affiliations:** Physics Department and Solid State Institute, Technion-Israel Institute of Technology, Haifa 32000, Israel.

## Abstract

Coherent manipulation of matter waves, a distinctive hallmark of quantum mechanics, is fundamental to modern quantum technologies. Spatial adiabatic passage (SAP) is a prime example of this phenomenon, where a wave packet is transferred between two uncoupled localized modes by adjusting the tunneling coupling to an intermediate third mode in a counterintuitive sequence. Although this concept was introduced over two decades ago, its observation was previously limited to electromagnetic waves. In this study, we demonstrate this quantum interference effect using massive particles that tunnel between three micro-optical traps (“optical tweezers”). We begin by preparing ultracold fermionic atoms in low vibrational eigenstates of one trap, followed by manipulating the distance between the traps to execute the SAP protocol. We observe a smooth and high-efficiency transfer of atoms between the two outer traps, with a very low population remaining in the central trap. These findings open possibilities for advanced control schemes in optical tweezer array platforms.

## Teaser

Atoms bypass central trap in three-trap chain via tunneling and quantum interference.

## INTRODUCTION

Modern quantum technologies require precise and rapid control of quantum states. Adiabatic following is one of the most effective methods for achieving this control by gradually connecting the initial and final states through a slow change of a specific parameter. In a two-level system coupled by an external field, continuously changing the drive frequency across the transition resonance leads to population inversion, a process known as adiabatic rapid passage (*1*). A well-known extension of this concept to a three-level system, in either a lambda or ladder configuration, is stimulated Raman adiabatic passage (STIRAP) (*2*). STIRAP enables the transfer of population between two of the three states by coupling them to an intermediate state with two driving fields. The unique aspect of STIRAP is the counterintuitive sequence of the driving fields, which keeps the system in a dark state involving only the initial and final states. This is particularly advantageous in atomic systems where the intermediate state has fast radiative decay. Over the years, STIRAP has found wide-ranging applications in physics, chemistry, and engineering (*3*).

A particularly intriguing generalization of STIRAP involves three localized spatial modes that are coupled by tunneling (*4*–*8*). This technique enables the transfer of a wave packet initially centered at the first mode to the third mode, with negligible population at the second mode throughout the process, despite the fact there is no direct coupling between the first and third modes. Known as spatial adiabatic passage (SAP), this process raises fundamental questions regarding the velocity of the transferred particle (*9*) and can be a valuable tool for efficiently relocating atoms. In spite of the fact that the theoretical proposal was presented many years ago, SAP has only been experimentally realized with light in photonic waveguides (*10*–*13*). A related concept, involving the transfer of matter waves between sublattices within an optical lattice, was recently demonstrated (*14*). However, the spatial adiabatic transfer of massive particles between spatially separated potential wells has not yet been achieved.

In this work, we successfully transport few fermionic atoms between micro-optical traps (“optical tweezers”) using the SAP. Our experiment takes advantage of recent technological advancements in the dynamical control of optical tweezers (*15*). Initially, we arrange three optical tweezers in a line, with the atoms confined to only one of the external tweezers. By precisely manipulating the distance between the tweezers, we demonstrate that the atoms can be efficiently transferred to the other external tweezer, while the probability of finding them in the central tweezer remains low. We investigate the tunneling rate between two adjacent tweezers and the relation between the tunneling coherence time and the SAP process fidelity.

## RESULTS

### SAP with optical tweezers

**Fig. 1.**
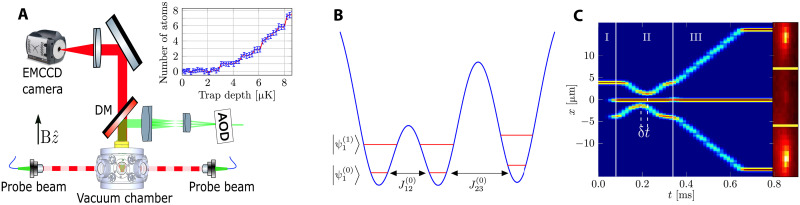
Experimental realization of SAP with optical tweezers. (**A**) Sketch of the experimental setup. Three tweezers are created by an AOD. The beams are then transmitted through an optical relay system and focused using an optical objective onto the plane of the atoms, which is perpendicular to both the axis of the magnetic field and gravity. The atomic fluorescence signal at a wavelength of 767 nm is collected with the same objective through a dichroic mirror (DM) and directed to an EMCCD camera. The inset shows the number of atoms versus the trap depth, as defined by the calculated difference between the local minimum and local maximum in the vicinity of the trap center. Each point is an average of 35 measurements, and the red line is a guide to the eye. The data were taken with a magnetic field gradient of 1.5 G/cm in the $\hat{z}$ direction. (**B**) Diagram illustrating three identical tweezers potentials, with two of them positioned closer to each other. (**C**) SAP sequence. The spectrogram of the rf signal applied to the AOD, converted to the position in the atom’s plane, is depicted. The sequence commences with the loading and preparation of tweezer 1 (I). Subsequently, tweezers 2 and 3 are turned on, and the counterintuitive sequence is executed (II). The atomic occupancy in each tweezer is measured using fluorescence imaging (III). An averaged image obtained from 500 runs is displayed on the right side.

Each of the three optical tweezers is generated by a far-off-resonance laser beam, creating a three-dimensional (3D) Gaussian potential through dipole interaction with the atoms (*16*). Because the beams propagate nearly parallel to each other, tunneling predominantly occurs in the radial direction, allowing us to effectively treat the system as 1D. The atoms occupy the lowest vibrational eigenstates of the tweezer potential, with all tweezers having the same beam parameters and depth. As the tweezer motion is adiabatic, tunneling only couples eigenstates with the same principal quantum number *n* at adjacent tweezers, denoted as $|\psi_{i}^{\left(n\right)}\rangle$ with *i* ∈ 1,2,3, and the initial state is $|\psi \left(t=0\right)\rangle =|\psi_{1}^{\left(n\right)}\rangle$ (see Fig. 1B).

The system evolves according to the time-dependent Schrödinger equation, with the Hamiltonian (*8*)

$$H\left(t\right)=\frac{\hbar }{2}\left[\begin{array}{ccc} 0 & J_{12}^{\left(n\right)}\left(t\right) & 0\\ J_{12}^{\left(n\right)}\left(t\right) & 0 & J_{23}^{\left(n\right)}\left(t\right)\\ 0 & J_{23}^{\left(n\right)}\left(t\right) & 0 \end{array}\right]$$ (1)

where $J_{\mathrm{ij}}^{\left(n\right)}$ represents the tunneling rate between the *n*th eigenstates in traps *i* and *j*, assumed here to be real. The Hamiltonian in Eq. 1 intentionally excludes direct tunneling between traps 1 and 3, in accordance with the SAP protocol. One particular eigenvector of interest with an eigenvalue of λ*_D_* = 0 is given by

$$|D^{\left(n\right)}\rangle =\text{cos}\theta^{\left(n\right)}|\psi_{1}^{\left(n\right)}\rangle -\text{sin}\theta^{\left(n\right)}|\psi_{3}^{\left(n\right)}\rangle$$ (2)

where $\theta^{\left(n\right)}\left(t\right)={\text{tan}}^{-1}\left[J_{12}^{\left(n\right)}\left(t\right)/J_{23}^{\left(n\right)}\left(t\right)\right]$ is the mixing angle (*8*). This state is referred to as “dark” in the context of the original STIRAP scheme as it does not include the excited state $|\psi_{2}^{\left(n\right)}\rangle$ . By adiabatically varying θ(*t*) from 0 to $\frac{\pi }{2}$ , the state ∣*D*^(*n*)^〉 evolves from $|\psi_{1}^{\left(n\right)}\rangle$ to $|\psi_{3}^{\left(n\right)}\rangle$ , allowing for the transport of an atom from tweezer 1 to 3 with no occupation of the middle tweezer.

Initially, the tweezers are far apart and the tunneling coefficients are zero. The counterintuitive pulse sequence begins by increasing $J_{23}^{\left(n\right)}$ to a certain value while keeping $J_{12}^{\left(n\right)}=0$ . This corresponds to θ^(*n*)^ = 0 and ∣*D*^(*n*)^〉 describing correctly the initial condition of all atoms being in tweezer 1. Then, $J_{12}^{\left(n\right)}$ is increased adiabatically while simultaneously decreasing $J_{23}^{\left(n\right)}$ (*8*). This sequence is commonly referred to as “counterintuitive” because the coupling is initially introduced between two empty tweezers. In our experiment, we control the tunneling rates by adjusting the distances between the tweezers. While the variation of the tunneling rate with distance depends on *n*, the SAP process can still be adiabatic for a wide range of *n* states (see the Supplementary Materials). Thus, we can perform the SAP experiment simultaneously with several atoms occupying different eigenstates.

### Experiment

In our experiment, we use fermionic ^40^K atoms prepared in the lowest vibrational states of one of the optical tweezers (see Materials and Methods). The tweezers are generated using a 1064-nm laser beam that passes through an acousto-optical deflector (AOD), giving us independent control over each trap’s position and potential depth (see Fig. 1A). An optical objective focuses these tweezers to a Gaussian waist of *w*_0_ = 1.15 μm.

The initially occupied tweezer is loaded from a Fermi gas with a temperature of *T*/*T_F_* ≈ 2, where *T_F_* is the Fermi temperature. The relative depths between the tweezer and the larger optical trap containing the Fermi gas ensure a nearly unit probability of occupying the lowest vibrational eigenstates of the tweezer (*17*). After loading the tweezer, we release the “reservoir” Fermi gas, and the magnetic field is adjusted to a point where the two relevant spin states of ^40^K are essentially noninteracting. To eliminate atoms in high-energy states, we reduce the tweezer’s intensity while simultaneously applying a magnetic field gradient (*17*, *18*). This procedure provides precise control over the average atom number within the tweezer, as depicted in the inset of Fig. 1A.

For SAP experiments, we turn on two additional tweezers, which remain unoccupied. During the SAP sequence, a magnetic gradient is applied to effectively nullify the average magnetic and gravitational forces experienced by the spins. The number of atoms and their position is determined by recording the fluorescence signal generated by two resonant, counterpropagating probe beams (*19*).

### Tunneling between two tweezers

**Fig. 2.**
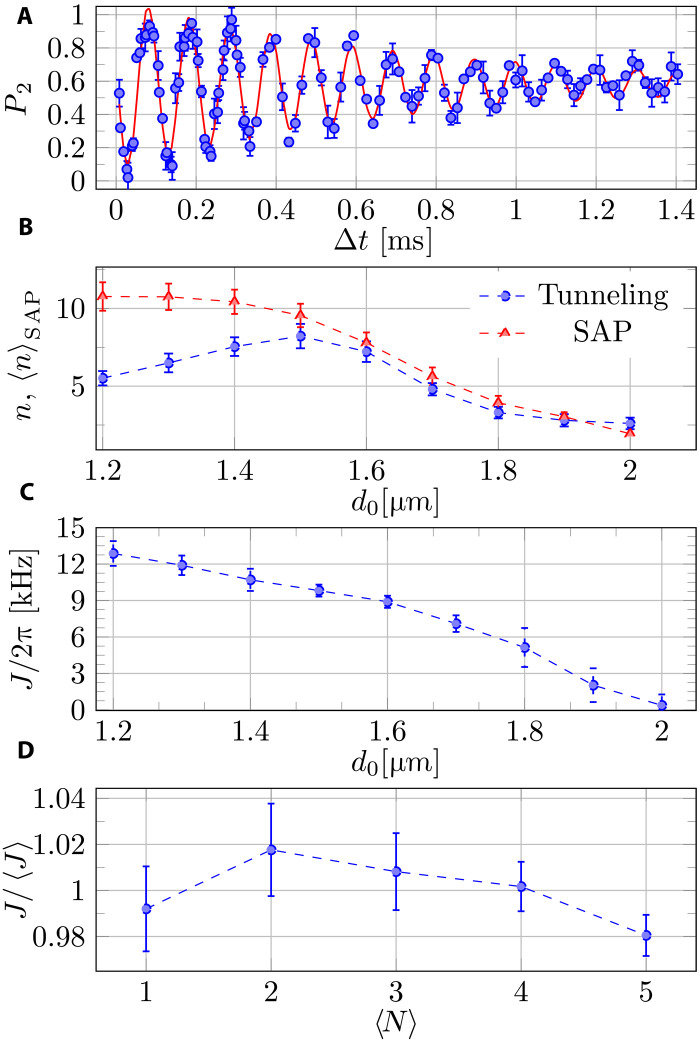
Tunneling measurements between two tweezers. (**A**) Relative population in the initially empty tweezer versus the waiting time, Δ*t*. The two tweezers are separated by a distance of *d*_0_ = 1.5 μm, and their depth is *U* = *k_B_* × 95 μK, resulting in a calculated radial trapping frequency of ω*_r_* = 2π × 38.9 kHz. The data are fitted with a decaying sine function, *P*_2_(*t*) = *c*_1_*e*^−*t*/τ^ sin (*Jt* + ϕ_0_) + *c*_2_ (red solid line), from which the tunneling frequency *J* and coherence time τ are extracted. (**B**) The number of coherent oscillations *n* = Jτ/2π is plotted against the distance between the centers of the tweezers, *d*_0_ (blue circles). In addition, we plot the time average of *n* during the SAP sequence, denoted as ${\langle n\rangle }_{\text{SAP}}=T^{-1}\int_{0}^{T}J\left(t\right)\tau \left(t\right)\mathrm{dt}$ (red triangles). (**C**) Tunneling rate as a function of *d*_0_. (**D**) The relative variation of the tunneling frequency versus the initial average number of atoms in the occupied tweezer, 〈*N*〉. These measurements were taken at *d*_0_ = 1.45 μm, and 〈*J*〉 is the average of *J* over *N*.

We first study tunneling between adjacent tweezers, which is a key aspect of the SAP process. We prepare one tweezer with a small number of atoms, as described earlier. Subsequently, we gradually turn on a second empty tweezer at a distance of *d*_0_ over a duration of 0.5 ms. After a certain waiting time Δ*t*, we move the traps away to a distance of 40 μm and then measure the relative population. As can be seen in Fig. 2A, slowly decaying coherent oscillations between the tweezers are observed. The decay is probably due to the involvement of multiple eigenstates, although tweezer intensity and position fluctuations may also contribute. Figure 2B depicts the number of coherent oscillations as a function of *d*_0_. We find an optimal distance of *d*_0_ = 1.5 μm, which corresponds to ~1.3 times the waist *w*_0_. The maximum number of coherent oscillations we observe is similar to the findings reported for Rb atoms (*20*).

The tunneling frequency as a function of *d*_0_ is presented in Fig. 2C. As expected, the frequency decreases as the distance increases. However, we observe a relatively weak dependence on the distance that deviates from our expectation based on numerical simulations of the 1D Schrödinger equation for the ground state wave function. This discrepancy could be attributed to deviations in the shape of the tweezer from an ideal Gaussian, the influence of 3D tunneling effects, and the population of higher vibrational states. Nonetheless, Fig. 2C provides valuable information regarding the relevant range of distances and the condition for adiabaticity in the SAP process. Furthermore, we conducted additional tunneling measurements with varying initial numbers of atoms, as depicted in Fig. 2D. The results demonstrate that the tunneling frequency is almost independent of the number of atoms, in the range of few atoms. This allows us to perform the measurement with few atoms simultaneously, thereby improving the signal-to-noise ratio.

### Demonstration of SAP between three tweezers

A schematic diagram illustrating the experimental SAP protocol is presented in Fig. 1C. The protocol initiates by preparing tweezer 1 with an average of three atoms. At *t* = 0, tweezers 2 and 3 are simultaneously turned on within 0.5 ms at a distance where the tunneling is negligible. All tweezers have a depth of 95 μK and a Gaussian waist of ω_0_ = 1.15 μm, identical to the tunneling measurements. In the counterintuitive sequence, tweezer 3 initiates motion toward tweezer 2, and after a delay of δ*t*, tweezer 1 follows the same trajectory toward tweezer 2. These trajectories modify *J*_12_(*t*) and *J*_23_(*t*), in accordance to the counterintuitive sequence. The position of each of the external tweezers is given by a Gaussian profile (*t* = 0 is the middle of the process)

$$x_{i}\left(t\right)=\pm x_{0}\pm \left(d_{\text{min}}-x_{0}\right)\text{exp}\left[-\frac{{\left(t\mp \delta t/2\right)}^{2}}{2\sigma^{2}}\right]$$ (3)

where *i* = 1,3 is the tweezer index, *x*_0_ = 4.5 μm is the initial position, *d*_min_ is the minimal distance to the central tweezer, and σ = 0.194*T* is the pulse width defined in terms of the total duration *T*. The upper (lower) sign pertains to tweezer *i* = 1 (*i* = 3). At any given time throughout the sequence, the population in all three tweezers can be measured by swiftly moving (≈15 μs) the two external tweezers back to their initial position where tunneling is essentially absent. This movement has been optimized to be faster than the tunneling dynamics while still maintaining a slow pace to prevent atom loss. Following this motion, the exact duration of the complete SAP sequence is awaited, after which the tweezers are moved again for imaging, as described earlier. The right panel of Fig. 1C presents an exemplary average fluorescence image of the three tweezers.

**Fig. 3.**
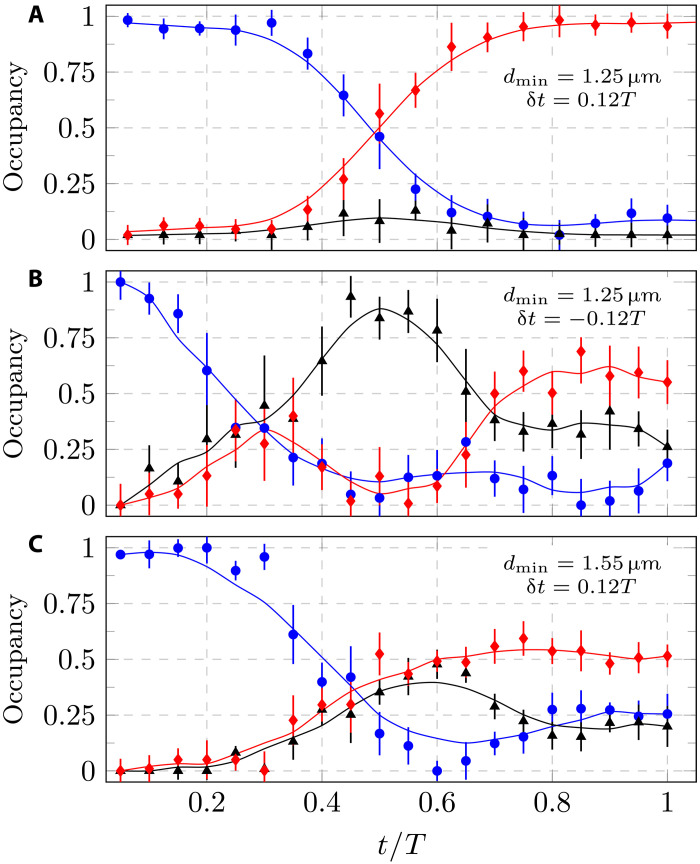
Measurements of SAP with three tweezers. The relative population in tweezer 1 (blue circles), tweezer 2 (black triangles), and tweezer 3 (red diamonds) is plotted at different times during the SAP sequence. The solid lines serve as a visual aid. In (**A**) and (**C**), we plot a SAP process with a counterintuitive profile (δ*t* > 0), while in (**B**), we use the intuitive sequence.

We present the main result of this paper, demonstration of a successful SAP scheme, in Fig. 3A. The sequence has a total duration of *T* = 0.25 ms, a delay of δ*t* = 0.12*T*, and a minimal distance of *d*_min_ = 1.25 μm. The data clearly illustrate the efficient transfer of atoms from tweezer 1 to tweezer 3 while maintaining a minimal probability of occupation in tweezer 2. This behavior exhibited insensitivity to the number of atoms within the range of one to six atoms (see the Supplementary Materials). For comparison, we conducted an experiment with identical parameters but using the intuitive pulse order, with δ*t* = −0.12*T*. The contrasting result, displayed in Fig. 3B, is remarkably different from the counterintuitive SAP depicted in Fig. 3A. In the middle of the sequence, there is nearly complete atom transfer to tweezer 2. While high transfer efficiency could potentially be achieved even in the intuitive pulse sequence, it would require fine-tuning the process parameter and would lack the robustness of the adiabatic SAP process (see the Supplementary Materials).

An interesting question is how the SAP process depends on the minimum distance between the tweezers. In Fig. 3C, we present the results of a similar experiment to that shown in Fig. 3A but with a larger minimum distance of *d*_min_ = 1.55 μm. Unexpectedly, in this case, the process is unsuccessful, leaving a substantial population in all three tweezers. This outcome may initially appear puzzling, considering that the largest number of coherent oscillations was achieved at this distance (Fig. 2B). However, during the SAP process, the distance between the tweezers changes. Consequently, a more appropriate measure would be to average the coherent tunneling oscillations over the entire SAP process. This quantity, represented by the red triangles in Fig. 2B, increases as the distance decreases. We have constrained our investigation to the regime *d*_min_ > *w*_0_ to ensure that there is a barrier between adjacent tweezers.

### SAP fidelity

To quantify the fidelity of the SAP process, we introduce the following function

$$f_{\text{SAP}}=P_{3}\left(T\right)\left[1-T^{-1}\int_{0}^{T}P_{2}\left(t\right)dt\right]$$ (4)

where *P_i_*(*t*) represents the probability of finding an atom in tweezer *i* at time *t*. This definition combines the requirement of high-fidelity transfer of the atoms to tweezer 3 and a low population in tweezer 2 throughout the process. In an ideal counterintuitive SAP process, *f*_SAP_ approaches unity.

**Fig. 4.**
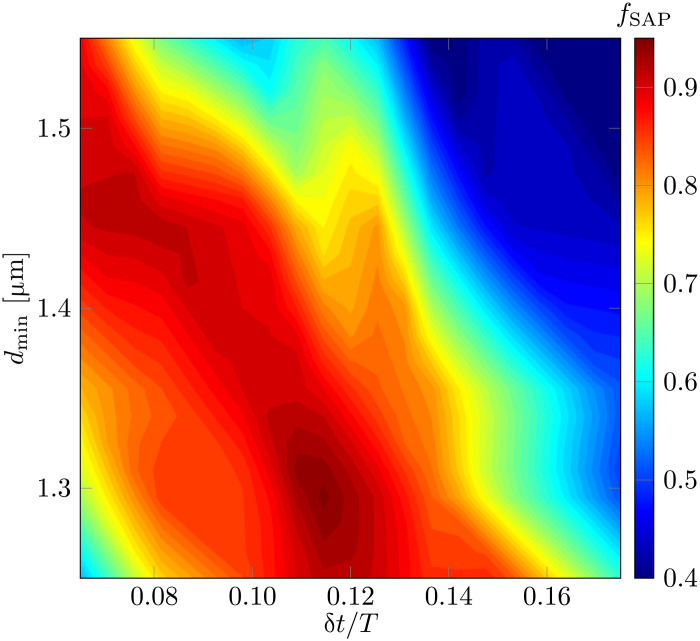
SAP fidelity. Mapping of the *f*_SAP_ versus the minimal distance, *d*_min_, and pulse delay, δ*t*. The data used in this figure were collected with a temporal resolution of 0.015*T* and a spatial resolution of 0.05 μm.

In Fig. 4, we show the fidelity of the SAP protocol as a function of the minimal distance between the tweezers and the delay time. A high-fidelity operation is achieved over a wide range of parameters, as expected in an adiabatic process. The optimal time delay is ~0.12*T*. For Gaussian temporal profiles of *J* with a width ξ, the theoretical optimal delay is approximately $\sqrt{2}\xi$ (*8*). Using our tweezer trajectory and the measured *J*(*d*_0_) (Fig. 2C), we calculate ξ/*T* ≈ 0.082. This value corresponds to an optimal delay of 0.116*T*, which closely aligns with our observation.

## DISCUSSION

In this work, we have demonstrated spatial adiabatic transfer of few ultracold fermionic atoms between three optical tweezers. Similar to other adiabatic control schemes, the key advantage of the SAP lies in its robustness to experimental imperfections and fluctuations in parameters. Our findings have revealed that the fidelity of the SAP process is ultimately limited by the coherence time of the tunneling dynamics. The Gaussian profiles of the tweezers lead to several important distinctions compared to ideal truncated Harmonic potentials. First, the potential depths of each tweezer vary depending on its distance to other tweezers. As the SAP process can be understood as the adiabatic following of a state connecting the initial and final states, the mutual influence mainly leads to increased population in the central trap. It may be possible to further enhance the SAP fidelity by dynamically compensating for the depth mismatch (*21*).

In our experiment, all atoms start localized in the same tweezer and are transported in parallel and in a similar manner to the destination tweezer. In a more general scenario, different atoms could be initially localized in different tweezers, and the adiabatic process could be engineered to transfer each to a different final state. If the atoms are noninteracting and start in orthogonal states, then they remain in orthogonal states throughout the adiabatic process. In this sense, they are “transparent” to one another, allowing for a high level of parallel control. Another interesting aspect of a multiatom adiabatic process is the effect of interactions, which could easily be tuned near a Feshbach resonance (*22*). Generally speaking, the interaction energy depends on the atomic density squared and thus introduces an inherent nonlinearity into the SAP problem. The adiabatic transport of a Bose-Einstein condensate was studied in (*23*–*25*).

The SAP protocol can be extended to encompass more than three tweezers (*26*, *27*). In the context of a chain of potential wells, the adiabatic passage exhibits characteristics reminiscent of Thouless pumping (*28*, *29*), enabling robust transfer of topological edge states (*30*, *31*). Therefore, the SAP has the potential to serve as an essential building block in tweezer array quantum technology platforms.

Beyond spatial transport, adiabatic following can be used to coherently split and recombine an atomic wave packet. In the case of splitting, this is achieved by designing a time-dependent potential to drive an adiabatic evolution from a state initially localized in one trap to a balanced superposition of two traps at the end. It has been recently proposed that these adiabatic atomic beam splitters and combiners could be implemented with optical tweezers and combined to form a fully guided atomic interferometer (*32*). A tweezer-based atomic interferometer could substantially extend the probing time beyond what current technology allows while also offering submicrometer positioning accuracy and almost complete flexibility in shaping the atomic trajectory. Similar to our SAP experiment, the splitting and recombining schemes can work simultaneously with many vibrational states, enabling multiatom interferometry in a single run.

## MATERIALS AND METHODS

The apparatus consists of a stainless steel chamber with two reentrant flanges with a 64-mm diameter viewports, located above and below (see Fig. 1A). These flanges allow placement of an optical objective with a numerical aperture (NA) of 0.75 at a distance of ~21 mm from the atomic plane. ^40^K atoms are dispensed in a separate glass chamber (*33*), where they are collected and guided into the main chamber using a 2D magneto-optical trap (MOT) (*34*). The cooling sequence continues by capturing the atoms with a 3D MOT, followed by gray molasses cooling (*35*) and degenerate Raman sideband cooling (*36*). The final cooling stage reduces the temperature to around 1 μK and optically pumps the atoms into the two lowest Zeeman eigenstates *m_F_* = −7/2,−9/2 of the *F* = 9/2 hyperfine manifold, with relative populations of 20 and 80%, respectively. The atoms are then loaded into a far-off-resonance crossed dipole trap (CDT) operating at a wavelength of 1064 nm with an aspect ratio of 1:2. After a brief optical evaporation period of 0.6 s, a radio frequency (rf) pulse is applied in a magnetic field of 185 G to make the spin mixture balanced. At this stage, the CDT holds ~80,000 atoms per spin state at approximately twice the Fermi temperature.

Subsequently, a single optical tweezer is turned on, overlapping with the Fermi gas. It is loaded with ~500 atoms per spin state, with nearly a unit probability of occupying the lowest eigenstates (*17*). The optical tweezers are generated using a 1064-nm laser beam that passes through an AOD, diffracting the beam to multiple angles based on the spectral content of its rf drive (see Fig. 1A). These diffracted beams are then optically guided to the optical objective through a set of telescopes that adjust its diameter. The position and intensity of each tweezer can be dynamically controlled by adjusting the rf drive. Once the tweezer is loaded, the CDT is gradually turned off, and the magnetic field is adjusted to its final value where the two spin states are almost noninteracting (208.7 G) (*37*).

To remove atoms in high-energy states, the intensity of the tweezer is reduced while also applying a magnetic field gradient of 2.5 G/cm along the $\hat{z}$ axis (*17*, *18*). Because of the difference in magnetic moment, the *m_F_* = −7/2 spin state typically has two more atoms. However, because the spins are noninteracting, this difference is irrelevant for the SAP process. The preparation sequence is completed by gradually increasing the power of the tweezer. During the SAP sequence, a magnetic gradient is set to the mean value required to cancel gravity for each of the two spin states, leaving ~5% of the gravitational potential, which is negligible compared to the depth of the tweezer. To measure the number of atoms in each tweezer, we reduce the magnetic field to 3 G and apply two counterpropagating laser beams resonant with the transition ∣*F* = 9/2〉 → ∣*F*′ = 11/2〉. These beams have a linear polarization perpendicular to the direction of the magnetic field, enabling them to drive σ_±_ transitions. To avoid interference and the formation of a standing wave pattern, the beams are turned on intermittently, each for a duration of 1 μs, with a total duration of 80 μs (*19*). The scattered photons are collected using the same high NA objective and directed onto an electron multiplying charge-coupled device (EMCCD) camera. Two calibration methods are used to determine the parameters of the tweezers: direct imaging of the beam to obtain its size and modulation of a piezoelectric actuator to measure the harmonic trapping frequency through atom loss. The measured values align with calculations based on beam power and size. The final potential depths of all three tweezers are equalized by ensuring that the long-term equilibrium population in each pair of adjacent tweezers, following a tunneling experiment like the one depicted in Fig. 2A, is identical.
