# Supplementary material for: Spatial adiabatic passage of ultracold atoms in optical tweezers
Source: Sci Adv. 2024 Oct 2;10(40):eadl1220. doi: 10.1126/sciadv.adl1220 (PMC11446269; doi:10.1126/sciadv.adl1220)
Supplement: Supplementary file 1 — Supplementary Text Figs. S1 to S3 [file sciadv.adl1220_sm.pdf]

Science Advances
AAAS

Supplementary Materials for

Spatial adiabatic passage of ultracold atoms in optical tweezers

Yanay Florshaim *et al.*

Corresponding author: Yoav Sagi, yoavsagi@technion.ac.il

Sci. Adv. **10**, eadl1220 (2024)
DOI: 10.1126/sciadv.adl1220

This PDF file includes:

Supplementary Text
Figs. S1 to S3

Supplementary Text

SAP with different number of atoms

The core principle of SAP is that the atom's state undergoes an adiabatic transition, starting from the first trap and concluding in the third trap. This transition happens by gradually altering certain parameters, such as the distance between the traps, as elaborated in the main text. This adjustment may enable the adiabatic following not only of the ground vibrational state of the first trap but also of its higher eigenstates. For this to occur, the modification must be sufficiently slow relative to the energy gaps in the spectrum of the relevant excited states. We illustrate this phenomenon through numerical simulations in Fig. S1, which demonstrates that the SAP process effective for $n = 0$ also successfully transfers a state initially at $n = 4$. The selection of $n = 4$ here serves merely as an example, with this behavior persisting across a broad spectrum of eigenstates.

Crucially, we have tested the SAP's sensitivity to the initial state in experiments. This was achieved by preparing the tweezer with varying average numbers of atoms and executing the same SAP sequence. The findings, depicted in Fig. S2, unequivocally indicate that the SAP sequence remains unaffected by the initial atom count, within a range of a few atoms.

Comparison between the counter-intuitive and intuitive pulse sequences

Fig. S3 presents results of numerical simulations intended to compare between the robustness of the intuitive and counter-intuitive pulse sequences. The simulation solves numerically the Lindblad master equation:

$$\dot{\rho} = -\frac{i}{\hbar}[H,\rho] - \gamma L\rho L^{\dagger},$$

Where the Hamiltonian is given by

$$H = \frac{1}{2}\hbar\begin{pmatrix} 0 & J_{12}(t) & 0 \\ J_{12}(t) & 0 & J_{23}(t) \\ 0 & J_{23}(t) & 0 \end{pmatrix}$$

And the decay is given by

$$\gamma L\rho L^{\dagger} = \frac{1}{2}\begin{pmatrix} 0 & \Gamma\rho_{12} & 0 \\ \Gamma\rho_{21} & 0 & \Gamma\rho_{23} \\ 0 & \Gamma\rho_{23} & 0 \end{pmatrix}.$$

The positions of the tweezers are defined as in Eq. (3) of the main text, while the tunneling rates $J_{12}(t), J_{23}(t)$ are evaluated directly from Fig.2(c) as a function of the distances between the tweezers.

The simulation shows that, in the long-time limit, high fidelity transfer of the atom from the first to the third trap using the counter-intuitive process is robust to decay and does not require fine-tuning of parameters. In contrast, the intuitive process is very sensitive both to the total duration and the decay.

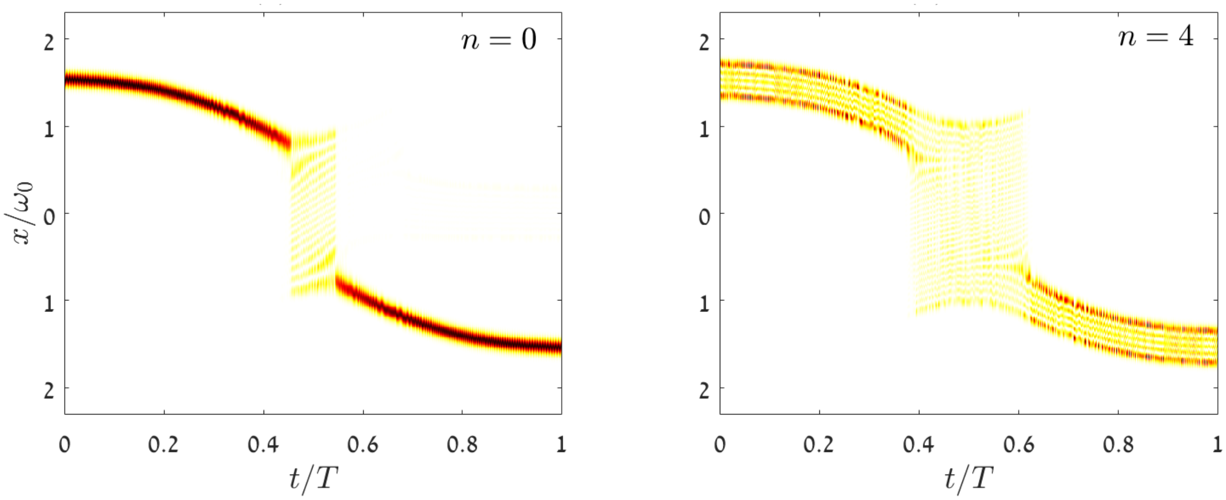

Fig. S1.

Numerical simulations of a SAP sequence. We solve Schrödinger's equation in 1D and plot the waveform evolution in space-time, where the colormap is proportional to $|\psi(x)|^2$. On the left, the atom is initialized at the ground state ($n = 0$), while on the right it is initialized at the fourth excited state ($n = 4$). Simulation parameters: trap depth of 95 μK, $\omega_0 = 1.15$ μm, $x_0 = 1.8$ μm, $d_{\min} = 1.18$ μm, $T = 65$ ms, $\delta t = 0.05T$, $\sigma = 0.194\,T$.

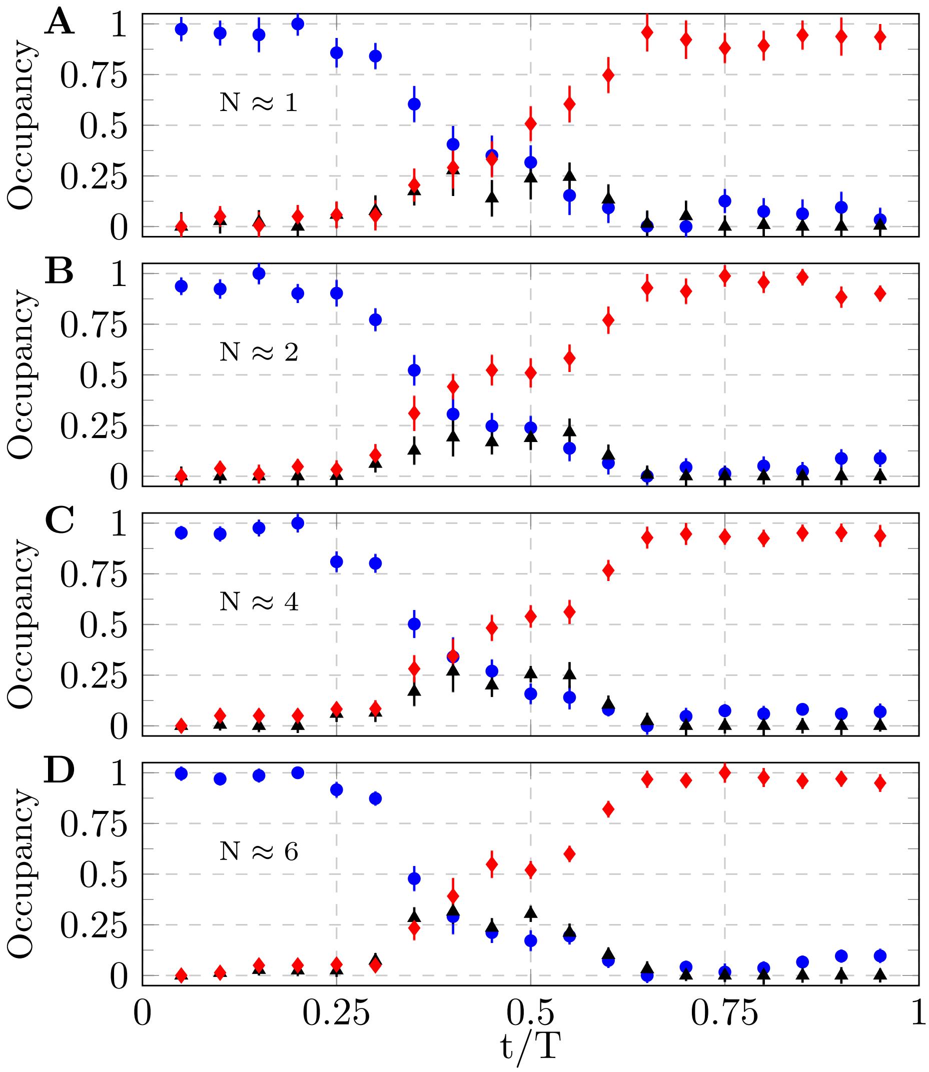

Fig. S2.

Experiments of SAP with different initial number of atoms in the tweezer. In this measurement, we changed the minimum trap power during evaporation in the preparation step leading to a different number of atoms in the initial state, N. Then, the two empty tweezers are turned on and the SAP sequence is executed. In these measurements, $\delta t = 0.1\,T$ ($T = 0.25$ msec) and $d_{\min} = 1.3$ µm.

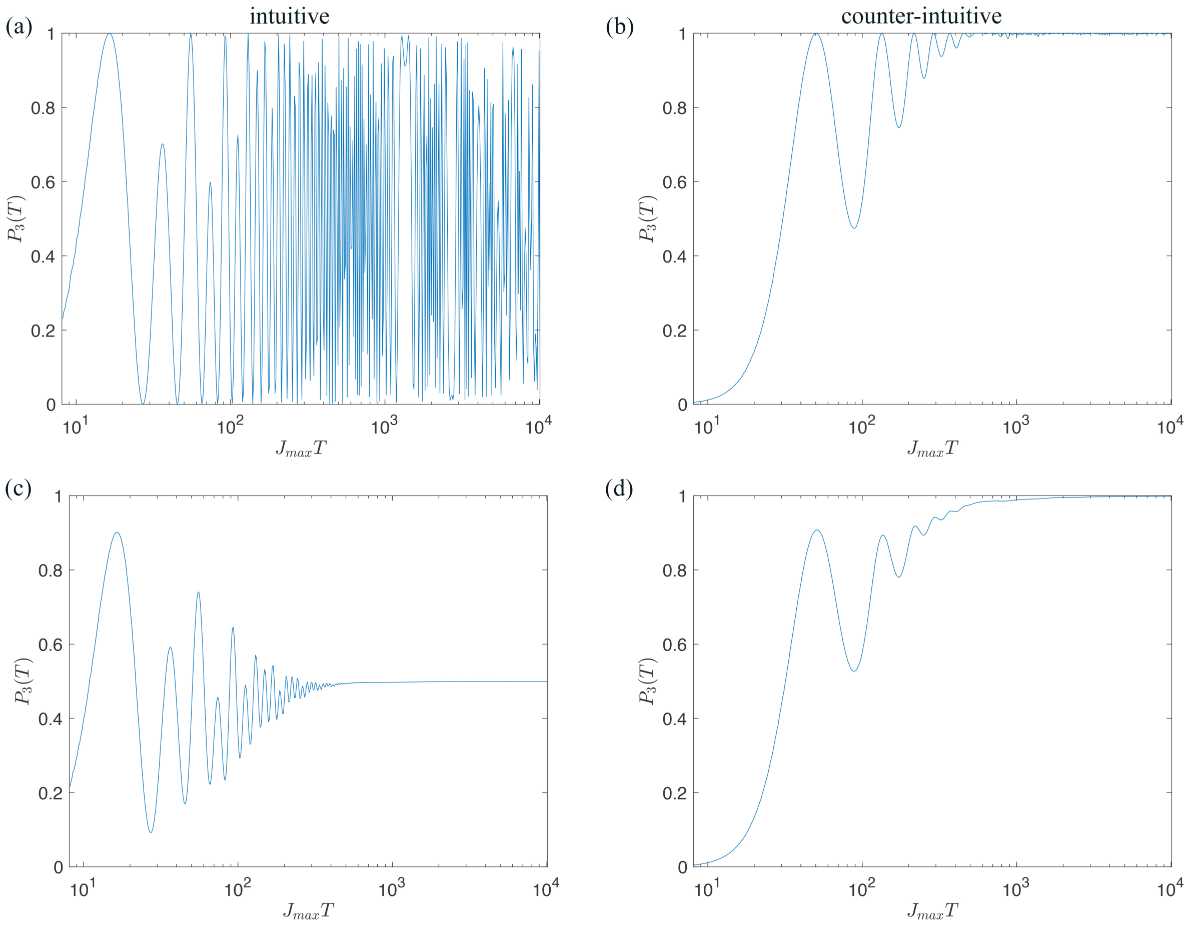

Fig. S3.

Comparison between the intuitive and counter-intuitive pulse sequences. We have simulated the tunneling between three traps with the intuitive (left-side panels) and counter-intuitive (right-side panels) pulse sequences, using a 1D Lindblad master equation without decay (panels a-b) and with decay of $\Gamma = 2\pi \cdot 1.6$ kHz (panels c-d). The probability to find the atom in the third trap, P_3, is plotted as a function of the total process duration, T, normalized by the maximal tunneling rate $J_{max} = 2\pi \cdot 12.87$ kHz.
